# *Chlorosis seedling lethality 1* encoding a MAP3K protein is essential for chloroplast development in rice

**DOI:** 10.1186/s12870-021-03404-9

**Published:** 2022-01-06

**Authors:** Jiayan Liang, Qiuxin Zhang, Yiran Liu, Jingjing Zhang, Wenyi Wang, Zemin Zhang

**Affiliations:** State Key Laboratory for Conservation and Utilization of Subtropical Agro-Bioresources, Guangdong Provincial Key Laboratory of Plant Molecular Breeding, College of Agriculture, South China Agricultural University, Guangzhou, 510642 China

**Keywords:** Rice, *OsCSL1*, Chloroplast development, MAP3K protein, Chloroplast-associated genes

## Abstract

**Background:**

Mitogen-activated protein kinase (MAPK) cascades are conserved signaling modules in eukaryotic organisms and play essential roles in immunity and stress responses. However, the role of MAPKs in chloroplast development remains to be evidently established.

**Results:**

In this study, a rice *chlorosis seedling lethality 1 (csl1)* mutant with a Zhonghua11 (ZH11, *japonica*) background was isolated. Seedlings of the mutant were characterized by chlorotic leaves and death after the trefoil stage, and chloroplasts were observed to contain accumulated starch granules. Molecular cloning revealed that *OsCSL1* encoded a MAPK kinase kinase22 (MKKK22) targeted to the endoplasmic reticulum (ER), and functional complementation of *OsCSL1* was found to restore the normal phenotype in *csl1* plants. The CRISPR/Cas9 technology was used for targeted disruption of *OsCSL1,* and the *OsCSL1*-Cas9 lines obtained therein exhibited yellow seedlings which phenocopied the *csl1* mutant. *CSL1*/MKKK22 was observed to establish direct interaction with MKK4, and altered expression of MKK1 and MKK4 was detected in the *csl1* mutant. Additionally, disruption of *OsCSL1* led to reduced expression of chloroplast-associated genes, including chlorophyll biosynthetic genes, plastid-encoded RNA polymerases, nuclear-encoded RNA polymerase, and nuclear-encoded chloroplast genes.

**Conclusions:**

The findings of this study revealed that *OsCSL1* played roles in regulating the expression of multiple chloroplast synthesis-related genes, thereby affecting their functions, and leading to wide-ranging defects, including chlorotic seedlings and severely disrupted chloroplasts containing accumulated starch granules.

**Supplementary Information:**

The online version contains supplementary material available at 10.1186/s12870-021-03404-9.

## Background

Chloroplasts are defined as organelles that play specific roles in the conversion of light energy to chemical energy via photosynthesis [1], and their functional and structural integrity are vital for normal plant growth and development [2, 3]. Abnormal chloroplast function is generally reflected in changes in leaf pigmentation [4, 5]. In rice, several pentatricopeptide repeat proteins (PPR) have been identified and are reportedly involved in chloroplast development, including *Young Seedling Albino (YSA)* [6], *Albino Seedling Lethality3 (ALS3)* [7], *Thermo-sensitive Chlorophyll-Deficient 10 (TCD 10)* [8], and *OsPPR6* [9]. Additionally, genes from several other families have been implicated in chloroplast development. For example, *Albino Leaf 1 (AL1)* and *AL2* encoding an octotricopeptide repeat protein and chloroplast group IIA intron splicing facilitator were identified, respectively [10, 11]; additionally, alterations in the number and structure of thylakoids resulted in the development of albino leaves and in the occurrence of seedling death at an early developmental stage in rice [10, 11]. Furthermore, rice *Young Leaf Chlorosis 2 (YLC2)* has been shown to encode the stroma-localized heme oxygenase 2, and seedlings of the *ylc2* mutant have been observed to exhibit a chlorotic phenotype and defective chloroplast structures [12]. Recently, *Glycinamide ribonucleotide synthetase (GARS)*, which catalyzes the second step in purine nucleotide biosynthesis, has been identified to be involved in chloroplast development in rice by affecting the expression of plastid-encoded genes [13].

Chloroplasts are semi-autonomous organelles containing a unique genome and gene expression system [14]. Precise chloroplast function is coordinately mediated by two types of RNA polymerase, namely the nuclear-encoded RNA polymerases (NEPs) and plastid-encoded RNA polymerases (PEPs) [15, 16], which are essential for the biogenesis of photosynthetically active chloroplast in plants [16, 17]. The NEP complex is encoded by *RPOTp* and *RPOTmp*, the knockout of which has been found to result in delayed chloroplast biogenesis [18, 19]. Apart from PEPs and NEPs, chlorophyll biosynthetic genes (CBGs) and nuclear-encoded chloroplast genes (NECGs) have also been established to be involved in chloroplast development.

Mitogen-activated protein kinase (MAPK) cascades are considered important mechanisms involved in the transmission of exogenous or developmental signals to target molecules, and are generally highly conserved in eukaryotes [20, 21]. These cascades are typically characterized by the sequential phosphorylation of MAPK kinase kinase (MKKK/MAP3K), MAPK kinase (MKK/MAP2K), and MAPK. In rice, MAPK signaling pathways have been shown be involved in eliciting defense responses against blight disease [22], rice blast [23], and brown planthopper infestation [24, 25], and also in the responses to multiple abiotic stimuli [26–30]. OsMPK6 has been identified as a negative regulator involved in the resistance to bacterial pathogens, the reduced expression of which enhances resistance to different races of *Xanthomonas oryzae* pv. *oryzae*, mediated via salicylic acid and jasmonic acid signaling pathways [31, 32]. Recent studies have also revealed the involvement of MAPK cascades in plant development. For example, in rice, the OsMKKK10–OsMKK4–OsMPK6 cascade has been established to positively control grain size and is negatively regulated by GRAIN SIZE AND NUMBER1 (GSN1) [33, 34]. Currently, however, much remains unknown regarding the role of MAPK cascades in chloroplast development.

In this study, characterization of a novel MAP3K gene involved in chloroplast development in rice was reported, designated *chlorosis seedling lethality 1 (csl1)*, the mutation of which has been reported to result in energy deficiency and premature seedling death. *OsCSL1* encodes a MAPK kinase kinase22 (MKKK22), which targets the endoplasmic reticulum (ER); furthermore, protein–protein interaction analyses revealed that OsCSL1/OsMKKK22 interaction with MKK4. Expression of MKK4 was reduced in the *csl1* mutant. Collectively, these observations indicate that the MKKK2–MKK4 signaling pathway potentially plays an important role in chloroplast development in rice.

## Results

### The *csl1* mutation results in the development of chlorotic seedlings and a lethal phenotype

**Fig. 1:**
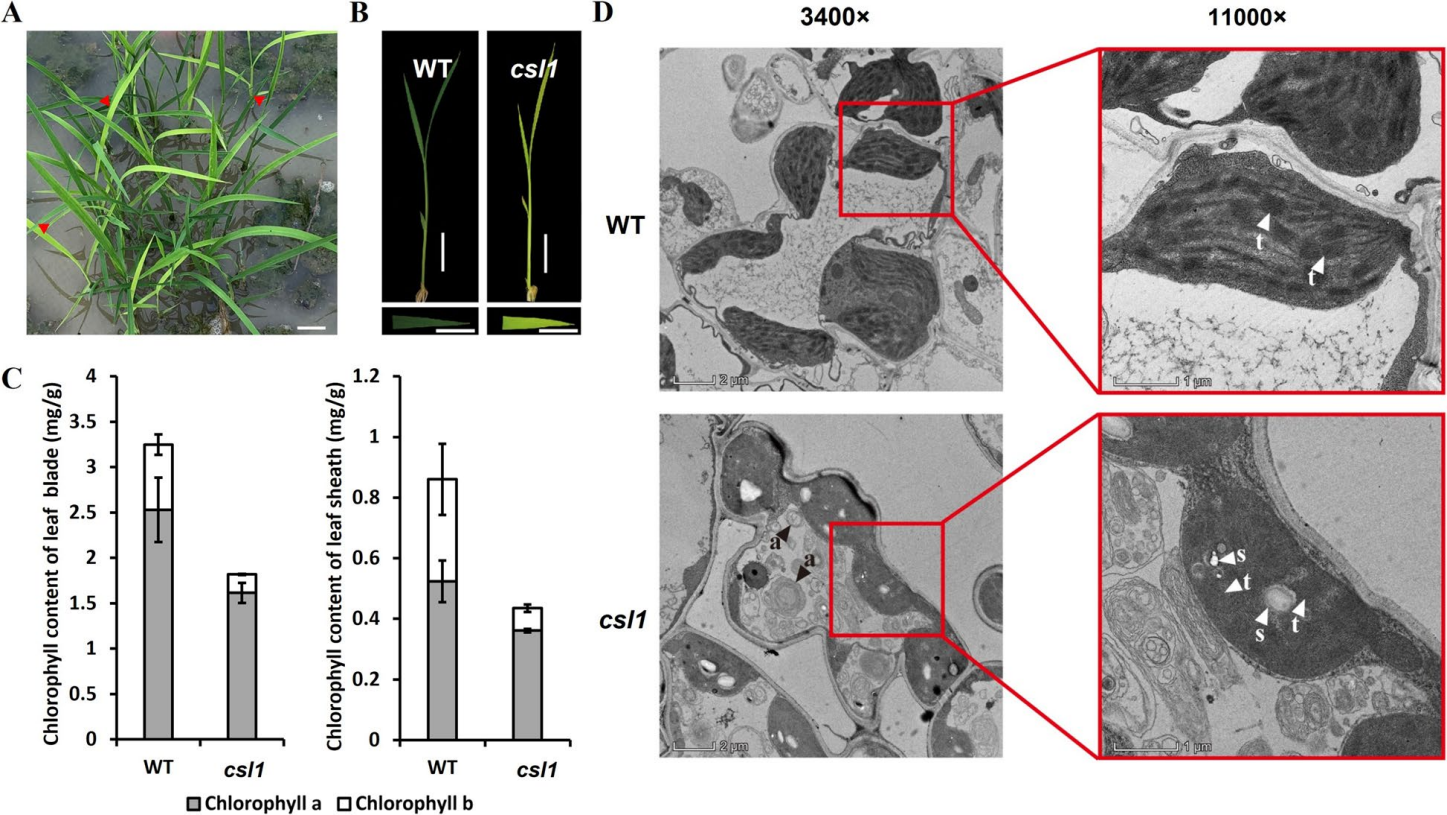
The phenotype of *csl1* mutant seedlings and transmission electron microscopy analysis. **A** Phenotypes of heterozygous progeny at the trefoil stage in a paddy field; *csl1* homozygous plants exhibited chlorotic seedlings (indicated using red arrows) compared with the green leaves of wild-type (WT) plants. **B** Phenotypes of WT (Zhonghua11) and *csl1* mutant at the trefoil stage; bars = 1 cm. **C** Chlorophyll content in the leaf blade (indicated in the left panel) and leaf sheath (indicated in the right panel) of WT and *csl1* mutant seedlings, Data are presented as mean ± S.E. **D** Transmission electron microscopy analysis of the leaves of WT and *csl1* mutant seedlings at the two-leaf stage. “a,” “t,” and “s” denote autophagosome, thylakoid, and starch granule, respectively; bars = 2 μm (magnification: 3400×) or 1 μm (magnification: 11000×)

To investigate the mechanisms underlying MAPK involvement in chloroplast development, a mutant, *chlorosis seedling lethality 1 (csl1)*, displaying defects in leaf pigmentation was identified among rice T-DNA insertion populations with a Zhonghua11 (*japonica*) background. Compared with the green leaves of wild-type (WT) plants, the *csl1* mutant exhibited a chlorotic seedling phenotype that developed prior to the trefoil stage (Fig. 1A, B). Unlike other chlorotic or albino leaf mutants, in which the leaf defects could be restored at later stages of development [19], *csl1* seedlings gradually withered and eventually died. To determine whether the observed chlorosis was associated with altered photosynthetic efficiency, chlorophyll contents in leaf blades and sheaths at the trefoil stage were further evaluated. The contents of chlorophylls *a* and *b* and total chlorophyll content in both leaf blades and sheaths were significantly reduced in the *csl1* mutant compared with WT seedlings (Fig. 1C), whereas no appreciable differences were detected between WT and heterozygous *csl1* lines (Fig. S1).

Chloroplasts are indispensable for normal plant growth and development, not only as an essential site for photosynthesis but also for a wide range of biochemical processes, including the synthesis of pigments, lipids, and hormones. Considering that *csl1* mutant seedlings were characterized by a yellow pigmentation and reduced chlorophyll contents, *csl1* mutation exerted an influence on chloroplast development was investigated. Transmission electron microscopy (TEM) observations of the chloroplast structures in *csl1* and WT seedlings at the two-leaf stage revealed that WT chloroplasts could be characterized by a distinct unidirectional thylakoid structure, whereas the intracellular structure of *csl1* mutant chloroplasts seemed to be disordered with indistinct thylakoids oriented in different directions, which accordingly contributed to a marked disruption of chloroplasts in the *csl1* mutant (Fig. 1D). Moreover, pronounced accumulation of starch granules in chloroplasts of the *csl1* mutant was detected (Fig. 1D). Collectively, these observations indicate that *OsCSL1* is essential for normal chloroplast development in rice.

### Molecular cloning of *OsCSL1*

**Fig. 2:**
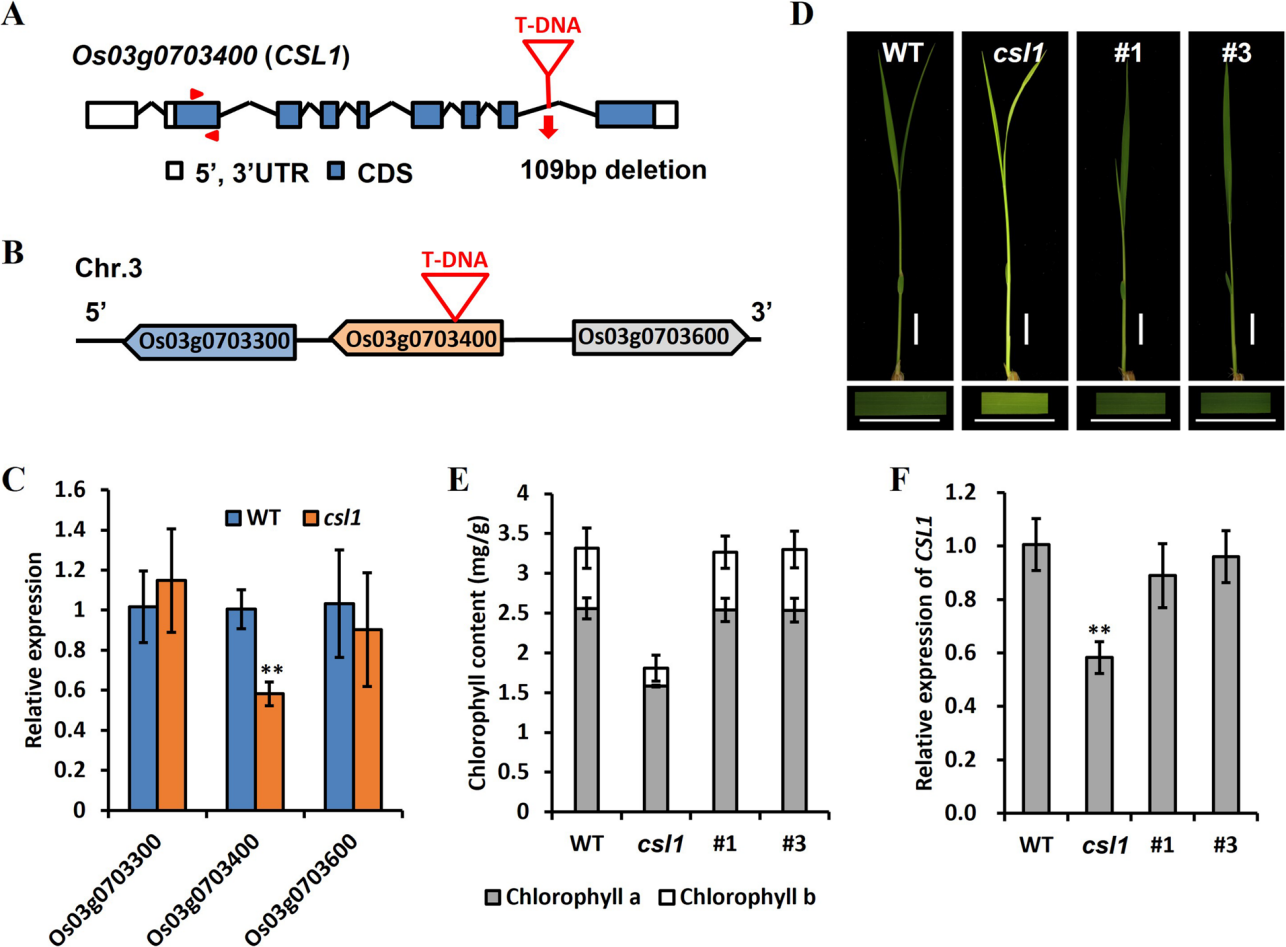
Identification of the *csl1* mutant and *OsCSL1* functional complementation of the *csl1* mutant. **A** Genomic organization of *OsCSL1* and T-DNA insertion in *the csl1* mutant. Boxes represent exons. **B** *Os03g0703300* and *Os0703600* are located upstream and downstream of the *OsCLS1* gene, respectively. **C** Relative expression levels of contiguous genes and *OsCSL1*. **D** Phenotypes of the wild-type (WT), *csl1* mutant, and complementary lines; bars = 1 cm. **E** Chlorophyll content in the WT, csl1 mutant, and complementary lines (**F**) Relative expression of *OsCSL1* in the WT, *csl1* mutant and complementary lines; ** *p*-value < 0.01, two-tailed, two-sample Student’s *t*-test. Data shown in D and E are presented as mean ± S.E

Genetic analysis indicated that the *csl1* phenotype could be subjected to control by a single recessive gene, as evidenced by the 3:1 segregation ratio between green and yellow seedlings (χ^2^ = 0.107, Table S1). To further characterize this gene, inverse polymerase chain reaction (IPCR) was performed to isolate the T-DNA genomic flanking regions in the c*sl1* mutant (Fig. S2). A BLAST search (https://rapdb.dna.affrc.go.jp/) revealed that the sequences of the flanking region were similar to those of a gene (*Os03g0703400*) predicted to encode a MAPK kinase kinase22 (MKKK22) located on rice chromosome 3, which has not been functionally characterized thus far. The coding region of *Os03g0703400* contains eight exons and seven introns (Fig. 2A). Notably, the full-length OsCSL1 protein consists of 489 amino acids, as opposed to the predicted 654 residues found in databases, the difference between which is attributable to a 165-amino acid deletion at the beginning of the first exon in the amplified product (Fig. S3).

The site of the T-DNA insertion is located between bases +4214 and +4322 in the seventh intron, leading to a 109-bp segment deletion in *Os03g0703400* (Fig. 2A). To eliminate interference from contiguous genes, expression levels of *Os03g0703300, Os03g0703400 (MKKK22/OsCSL1)*, and *Os03g0703600* were examined (Fig. 2B). RT-qPCR analysis revealed no appreciable differences between WT and *csl1* mutant plants with respect to the levels of *Os03g0703300* and *Os03g0703600* transcripts (Fig. 2C); however, the level of *Os3g0703400 (OsCSL1)* was found to be significantly reduced in the *csl1* mutant compared with the WT (Fig. 2C). Collectively, these findings indicate that *Os3g0703400* is a strong candidate for the *OsCSL1* gene.

### Mutation of *OsCSL1* is responsible for the development of a chlorotic seedling phenotype

To verify whether the chlorotic seedling phenotype was associated with a defective *Os03g0703400 (OsCSL1)*, genetic complementation assay using heterozygous *csl1*/*OsCSL1* plants was performed, owing to the lethality of the *csl1* mutation in homozygous seedlings. A ~9-kb WT genomic DNA sequence, comprising a ~2.5-kb sequence upstream of the start codon, a ~5.5-kb open reading frame, and a ~1-kb sequence downstream of the stop codon, was cloned into a binary vector and transformed into heterozygous *csl1*/*OsCSL1* plants. Positive transgenic plants derived from the *csl1*/*csl1* background were obtained, and used for further analyses. As shown in Fig. 2D, *csl1* seedlings characterized by a defective chlorotic phenotype were rescued by the *OsCSL1* transgene (Fig. 2D). Moreover, the expression of *OsCSL1* and chlorophyll contents in the transgenic seedlings recovered to normal levels (Fig. 2E, F). These observations thus revealed that the chlorotic phenotype of *csl1* mutant seedlings was associated with a defective *Os03g0703400*, thereby indicating that *OsCSL1* was the causal gene and it played an essential role in the regulation of rice leaf pigmentation.

**Fig. 3:**
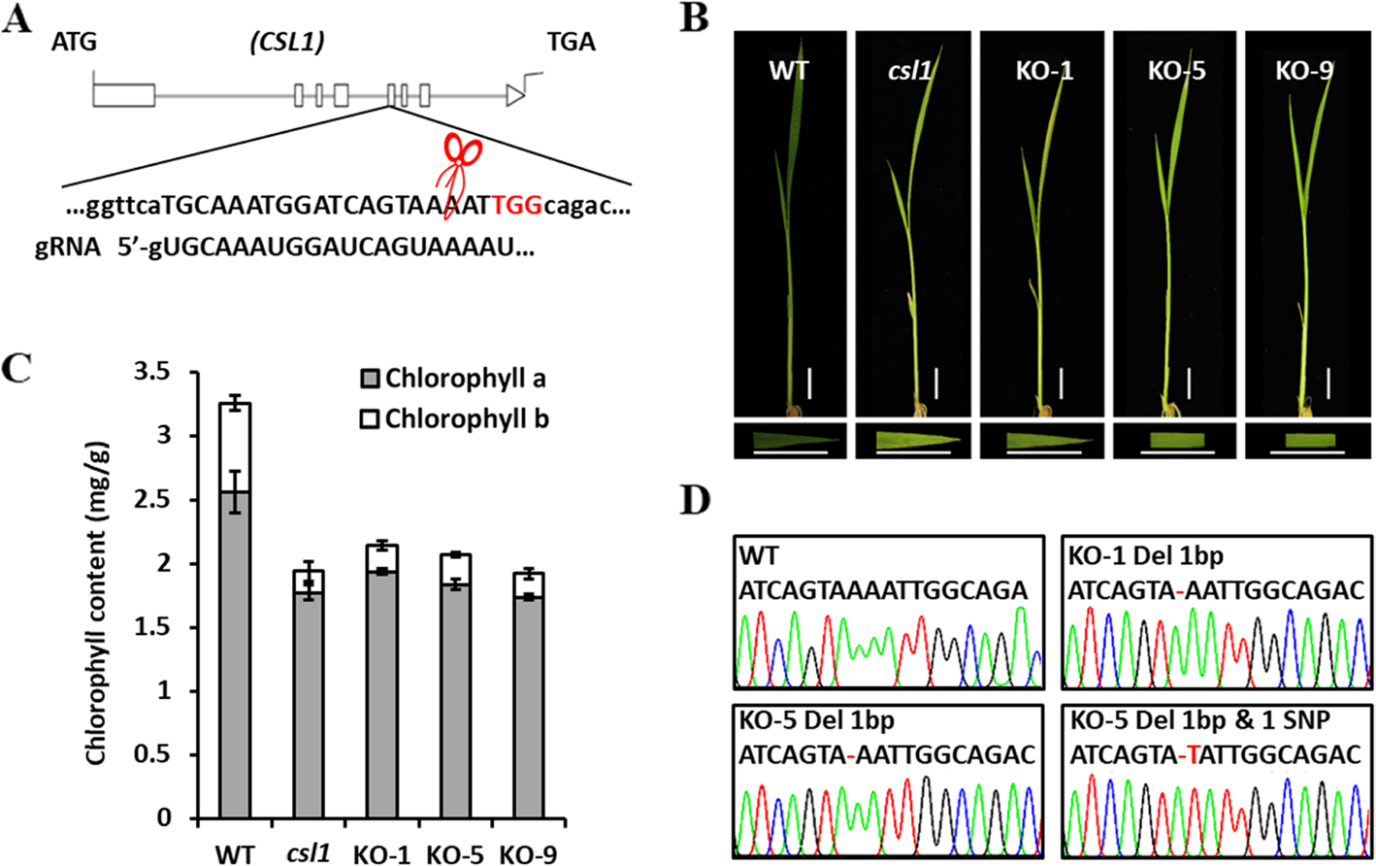
Characterization of *OsCSL1*-Cas9 lines. **A** A schematic representation of knockout target sites of *OsCSL1*. **B** Phenotypes of the wild-type (WT), *csl1* mutant, and *OsCSL1*-Cas9 lines; bars = 1 cm. **C** Chlorophyll content in the WT, *csl1* mutant, and *OsCSL1*-Cas9 lines. Data are presented as mean ± S.E. **D** *OsCSL1*-Cas9 lines containing insertion and deletion mutations were confirmed via sequencing

To further examine the molecular function of *OsCSL1* in rice, *OsCSL1* knockout mutant lines in the Zhonghua11 background were generated using the CRISPR/Cas9 genome editing technique. Accordingly, a series of transgenic *OsCSL1*-Cas9 lines were generated using a target site located in the fifth exon (Fig. 3A). Subsequent phenotypic observations revealed that the seedlings of *OsCSL1*-Cas9 lines presented with a chlorotic phenotype prior to the trefoil stage (Fig. 3B), which was similar to that of the *csl1* mutant. Consistently, similar to the findings documented with the *csl1* mutant, chlorophyll contents (chlorophyll *a*, chlorophyll *b*, and total chlorophyll) in the *OsCSL1*-Cas9 line seedlings were reduced compared with those in the WT plants (Fig. 3C). Moreover, the findings revealed that deletion and/or substitution events occurred in the *OsCSL1*-Cas9 lines (Fig. 3D); for example, *OsCSL1*-Cas9-1 and *OsCSL1*-Cas9-5, which contained a 1-bp deletion, and *OsCSL1*-Cas9-9, containing both deletions and substitutions (Fig. 3D). These findings indicated that *OsCSL1* knockout plants phenocopied the T-DNA insertional mutant, and mutation of the *OsCSL1* gene resulted in the development of the chlorotic seedling phenotype in rice.

### OsCSL1 is targeted to the endoplasmic reticulum

**Fig. 4:**
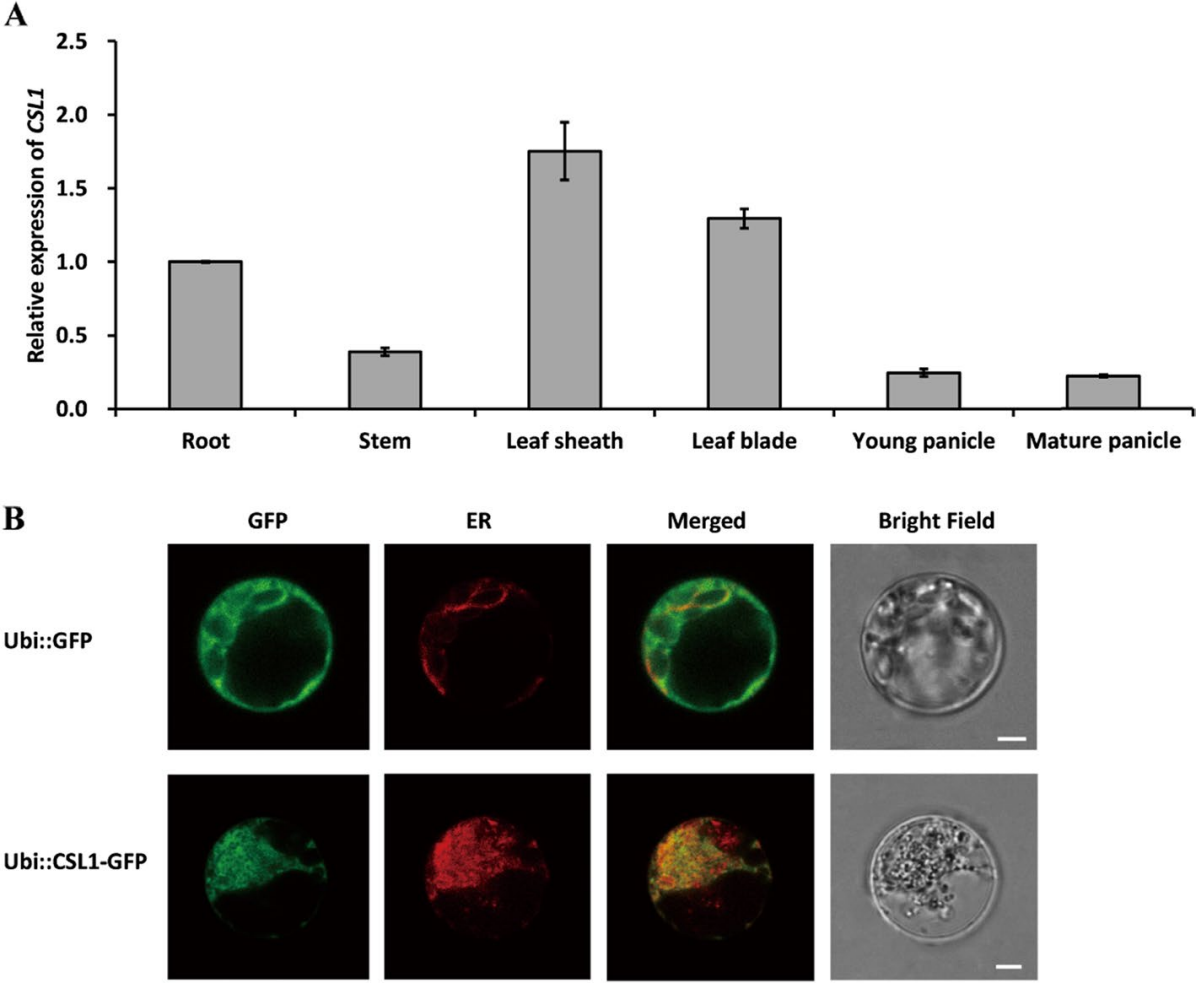
Expression pattern of *OsCSL1*. **A** Expression levels in different organs. **B** Subcellular localization of the OsCSL1-GFP protein in rice protoplasts

To elucidate the temporal and spatial expression of *OsCSL1*, RT-qPCR to investigate the patterns of expression in different tissues was performed, including root, stem, leaf sheath, leaf blade, young panicle, and mature panicle. The results revealed that *OsCSL1* was constitutively expressed in different tissues and organs, demonstrating markedly high levels in the leaf sheath and blade (Fig. 4A), and was weakly expressed in other tissues, such as the roots, stems, and young and mature panicles (Fig. 4A). These results thereby highlight the role of *OsCSL1* in plant development, particularly, in the developmental stages in the leaves.

To examine the subcellular localization of OsCSL1, a CSL-GFP (green fluorescent protein) fusion protein was transiently expressed in rice protoplasts. An OsCSL1-GFP fusion protein under the control of the *Zea mays* ubiquitin promoter was assembled and subsequently transformed into rice protoplasts. Laser scanning confocal microscopy of the transformed protoplasts revealed an overlap of the green signals generated by OsCSL1-GFP, and the red signals derived from ER-mCherry [35], observed as an orange coloration (Fig. 4B), thus indicating that OsCSL1 was located in the ER.

### Altered expression of chloroplast-related genes is observed in the *csl1* mutant

**Fig. 5:**
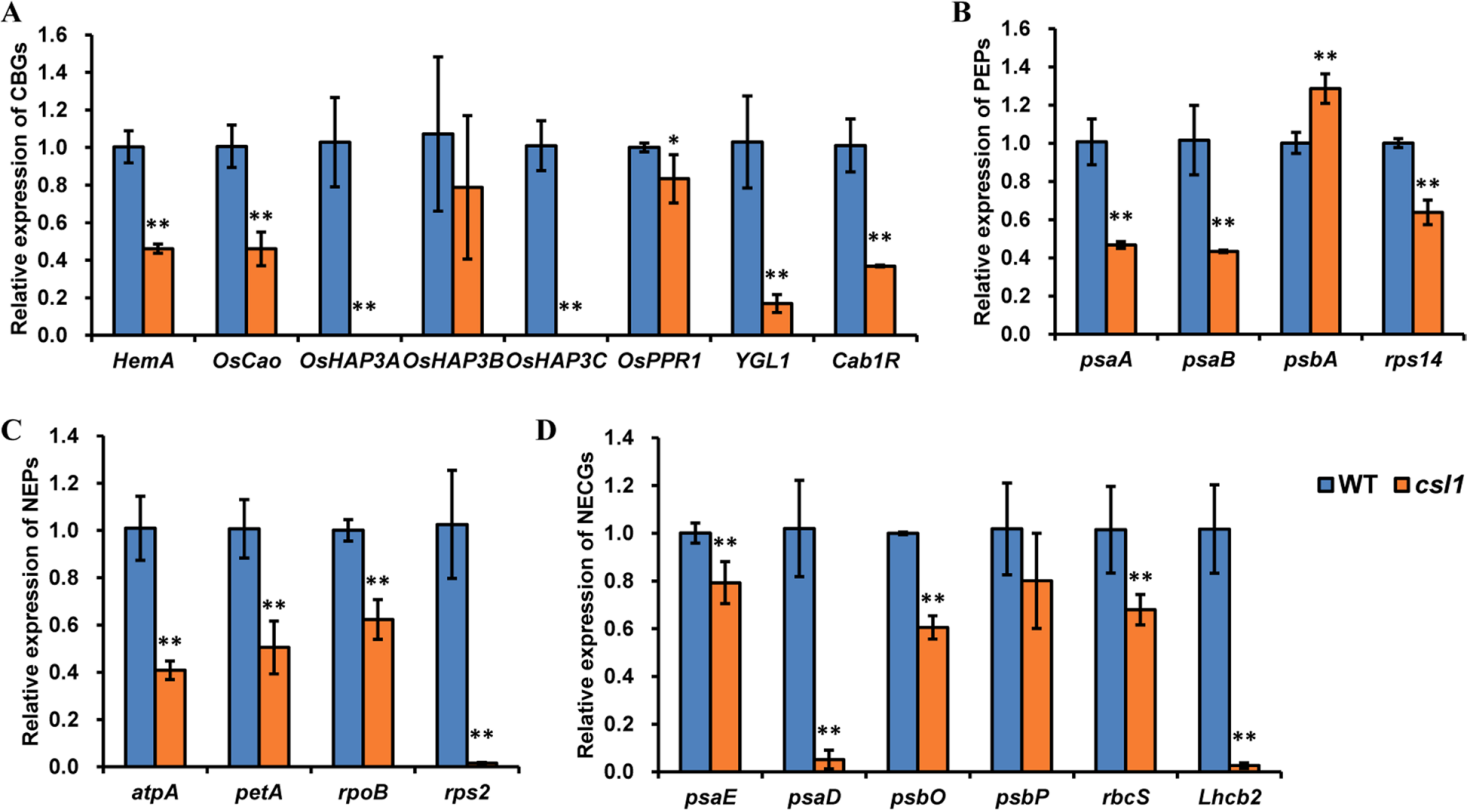
Expression of chloroplast-associated genes. Relative expression of chlorophyll biosynthetic genes (CBGs) (**A**), plastid-encoded RNA polymerases (PEPs) (**B**), nuclear-encoded RNA polymerases (NEPs) (**C**), and nuclear-encoded chloroplast genes (NECGs) (**D**). Data are presented as mean ± S.E., ** *p*-value < 0.01, two-tailed, two-sample Student’s *t*-test

Considering abnormal chlorophyll metabolism and plastid development in the *csl1* mutant, the expression of chloroplast development- and photosynthesis-related genes were investigated, including chlorophyll biosynthetic genes (CBGs), plastid-encoded RNA polymerases (PEPs), nuclear-encoded plastid RNA polymerase (NEPs), and nuclear-encoded chloroplast genes (NECGs). In line with expectations, significant reductions in the expression levels of several *CBGs* in the *csl1* mutant were detected, including those of *OsHAP3, HemA*, *OsCAO*, *YGL1*, and *Cab1R,* which were reduced by at least a 50% compared with those noted in the WT. Notable among these was the reduced expression of *OsHAP3A* and *OsHAP3C*, which were barely detectable in the *csl1* mutant (Fig. 5A). Furthermore, examination of the expression of PEPs, NEPs, and NECGs revealed that, with the exception of *psbA* and *psbP*, the expression levels of all investigated genes were significantly impaired in the *csl1* mutant (Fig. 5B-D). These findings accordingly provide evidence to indicate that chloroplast development, chlorophyll synthesis, and photosynthesis are potentially disrupted in the *csl1* mutant.

### *OsCSL1* establishes interaction with MKK4 and alters the expression of *OsMKKs* in the *csl1* mutant

**Fig. 6:**
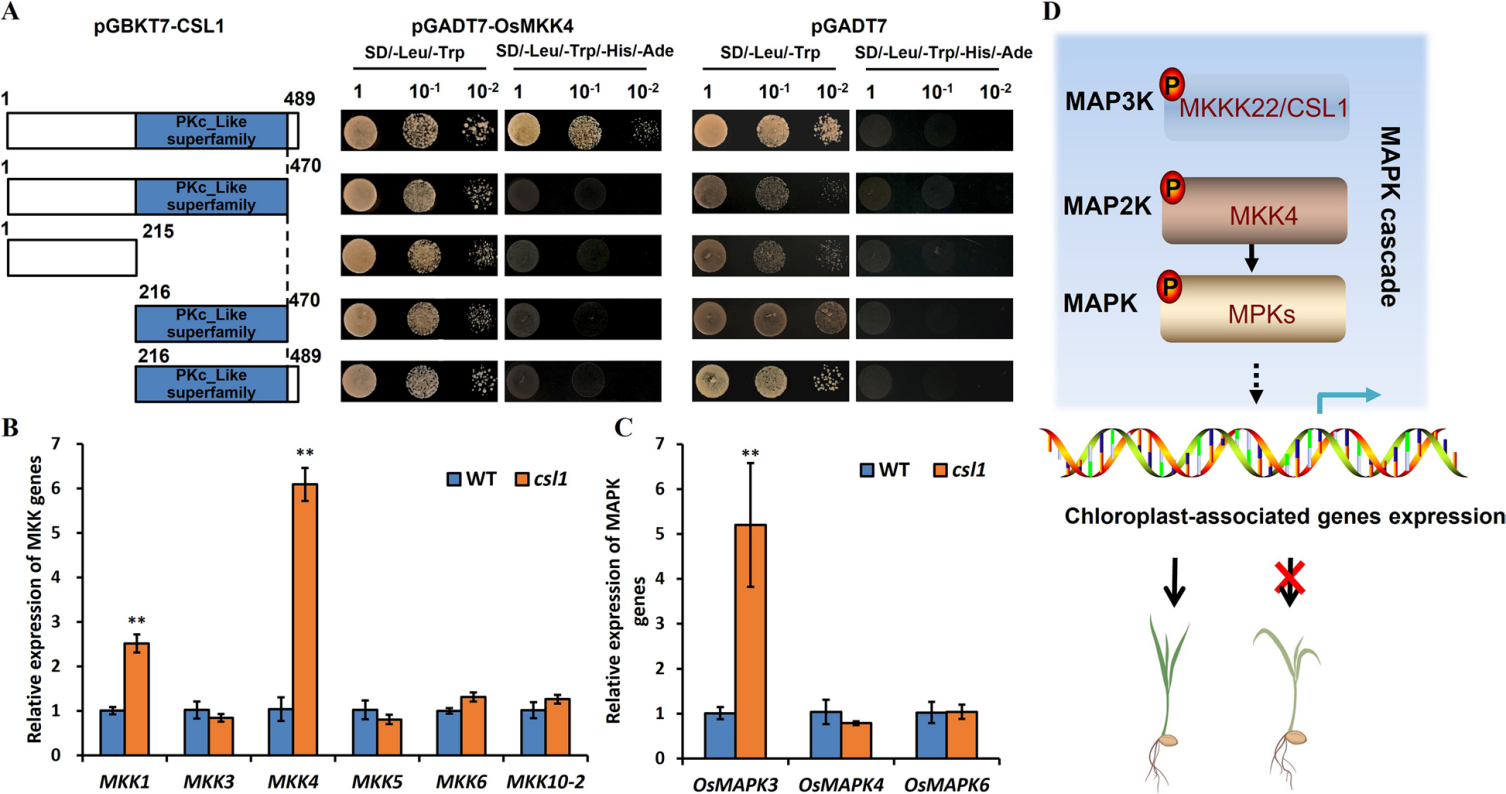
The interaction between OsCSL1 and MKK4 and the expression of mitogen-activated protein-related genes. **A** The interaction between OsCSL1 and MKK4 in a yeast two-hybrid assay. Schematic representation of full-length and truncated OsCSL1. The bait (BD) vector contained full-length or truncated *OsCSL1*, the prey (AD) vector harbored MKK4. Yeast strains were cultured on SD (/-Leu/-Trp) and QDO (-Ade/-His/-Leu/-Trp) selection media for 3 days. An empty prey (AD) vector was used as a negative control. **B** Relative expression of mitogen-activated protein kinase (MKK) genes. **C** A proposed model of OsCSL1/MKKK22 involved in chloroplast development and leaf color regulation in rice. The OsCSL1/MKKK22-MKK4 pathway potentially regulates the expression of chloroplast-associated genes, thereby affecting their function

In the MAPK cascade, MAP3K catalyzes the phosphorylation of MKK proteins, which subsequently leads to the phosphorylation of MAPKs and results in the stimulation of downstream target genes. Previously, yeast two-hybrid (Y2H) and protein microarray analyses have revealed the interactions established between MKK and MPK pairs and among a range of MAPKs and potential substrates [36, 37]. Thus, in the present study, Y2H assays were used to examine the interactions between *OsCSL1*/MKKK22 and MKKs, using either full-length or truncated OsCSL1 as baits for assessments of protein interactions. The Y2H results revealed that OsCSL1 interactions with OsMKK4 (Fig. 6A), and that this interaction was dependent on the presence of a full-length OsCSL1 (Fig. 6A). Further analysis of the expression of *OsMKK* genes in *csl1* mutant and WT plants revealed no significant differences in the expression of *OsMKK3*, *OsMKK5*, *OsMKK6*, or *OsMKK10-2* between WT and the *csl1* mutant (Fig. 6B); in contrast, a pronounced accumulation of *OsMKK1* and *OsMKK4* transcripts in the *csl1* mutant was detected (Fig. 6B). Considering that it has been previously reported that OsMKK1 and OsMKK4 potentially establish interactions with OsMAPK3, OsMAPK4, and OsMAPK6 [33, 38], the expression of *OsMAPK3, OsMAPK4,* and *OsMAPK6* were further investigated, and accordingly observed that the expression of O*sMAPK3*s was significantly higher in the *csl1* mutant than that in the WT (Fig. 6C). Taken together, these findings indicate the potential role of the MKKK22–MKK4 pathway in rice chloroplast development.

## Discussion

In plants, events occurring during the early seedling phase are essential determinants of long-term growth and development [39]. In the present study, a homozygous *chlorosis seedling lethality 1 (csl1)* mutant was characterization using T-DNA insertion lines with a rice cultivar Zhonghua11 *(O. japonica)* background. *csl1* seedlings were characterized by the development of yellow leaves (Fig. 1A) and occurrence of premature death after the trefoil stage. Inverse PCR analysis, used to identify DNA sequences flanking the T-DNA insertion region in the *csl1* mutant, indicated that *Os03g0703400*, encoding OsMKKK22, was a strong candidate for the *csl1* gene (Fig. 2A-C).

MAP3Ks are vital components of MAPK cascades, a group of signal transduction pathways that tend to be highly conserved in eukaryotic organisms [38]. Generally, MAPK cascades consist of three types of functional protein kinases, namely MAP3Ks, MAP2Ks, and MAPKs. MAP3Ks activate downstream MAP2Ks, which subsequently activate MAPKs that in turn target different cytoplasmic and nuclear effector proteins. Numerous studies have revealed that MAP3Ks are extensively involved in the generation of responses of plants to biotic and abiotic stresses, as well as hormonal signal transduction. For example, *GhMAP3K40* is reportedly associated with a reduced tolerance to biotic and abiotic stresses in *Nicotiana benthamiana* via negative regulation of plant development [40]. Additionally, *GhRaf19*, a Raf-like MAPKKK gene, has been demonstrated to negatively regulate the development of tolerance to drought and salt stress in cotton, and to positively regulate the development of resistance to cold stress [41]. Notably, mutation of *csl1*, encoding a MAP3K protein in rice, was associated with a lethal yellow seedling phenotype, and that chlorophyll contents in both the leaf blades and sheaths of *csl1* mutant seedlings at the trefoil stage were significantly lower than those in the WT (Fig. 1). To verify whether the observed *csl1* mutant phenotype was associated with *MAP3K*, complementation assays and knockout experiments were performed, the results of which indicated that the development of the lethal yellow seedling phenotype in rice *csl1* seedlings was attributable to mutation of the *Os03g0703400* gene (Figs. 2 and 3).

As mentioned previously, most studies reported in this field have highlighted the roles of MAP3Ks in plant adaptation to environmental conditions and as a regulator of signaling. For example, CONSTITUTIVE TRIPLERESPONSE 1 (CTR1) and ENHANCED DISEASE RESISTANCE 1 (EDR1), two of the best-studied MAP3Ks in plants, are known to be involved in the ethylene-mediated signaling response. Recent studies have revealed that MAP3Ks play important roles in plant growth and development. In this regard, it has been observed that compared with WT plants, transgenic *Arabidopsis* plants expressing MAP3Kδ4 are characterized by earlier bolting and more vigorous growth [42]; furthermore, overexpression of *MAP3K18* has been found to be associated with a smaller phenotype in *Arabidopsis*, demonstrating the promotion of senescence of the rosette leaves in transgenic plants compared with those of WT plants [43]. Nevertheless, the molecular mechanisms underlying the MAP3K-mediated regulation of plant growth and development, particularly leaf pigmentation, remain to be determined.

MAPK pathways are triggered in response to a diverse range of external stimuli [44], with the activation of the MAPK cascade leading to the phosphorylation of specific targets, including transcription factors, cytoskeletal proteins, and phospholipases [45]. In this regard, Y2H assay results indicated that OsCSL1 established interactions with OsMKK4 (Fig. 6A), and the expression of *OsMKK1* and *OsMKK4* was significantly increased in the *csl1* mutant compared with that in the WT (Fig. 6B). It has previously been established that *OsMKK1* is induced by salt stress, and that a high expression of this gene and its downstream target *OsMAPK4* contributes to enhanced seedling survival by increasing the transcript levels of *OsDREB2B* and *OsMYBS3* [38]. In rice, *OsMKK4/SMG1* plays multiple roles, among which the *OsMKK4–OsMPK3* and *OsMKK4–OsMPK6* cascades are implicated in the development of rice immunity [46]; additionally, the OsMKK4–OsMPK1/OsMPK6 cascade and downstream transcription factor *OsWRKY53* are involved in the rice wound response [47]. Furthermore, the findings of a recent study have revealed that the OsMKKK10–OsMKK4–OsMPK6 cascade leads to the coordination of a trade-off between grain number per panicle and grain size, and that this coordination is controlled by the negative regulator GSN1 [33], thereby indicating that MKK4 may function as a vital regulator of plant growth and development.

Chloroplasts are semi-autonomous organelles that are recognized as sites of photosynthesis in eukaryotic cells [48]. During early seedling development, proplastids are converted to chloroplasts via thylakoid biogenesis to facilitate energy fixation and metabolite production [39]. Compared with WT plants, discrete thylakoid structures were rarely detected in the chloroplasts of *csl1* mutant plants (Fig. 1D), and consistently, the abnormal leaf pigmentation of these plants indicated the presence of defective chloroplasts. Several mutants displaying variegated, pale-green, and albino leaves have been identified in different plant species [10, 19]. Reductions in the chlorophyll contents of leaf blades and sheaths at the trefoil stage in homozygous *csl1* mutants (Fig. 1) provided evidence to indicate that the premature death of mutant seedlings could be attributable to abnormal chloroplast development and reductions in chlorophyll biosynthesis.

In higher plants, chloroplast differentiation and development involves the following three principal steps: (i) the activation of plastid DNA synthesis, (ii) an increase in chloroplast number, and (iii) synthesis of the photosynthetic apparatus. Based on the assumption that abnormal chloroplast development during the early stages of plant growth could be attributable to the suppression of expression of chlorophyll biosynthetic, plastid, and nuclear genes, expression patterns of a series of chloroplast-related genes were analyzed, and accordingly found that the expression of genes involved in chlorophyll biosynthesis and chloroplast development, including those of *CBGs*, *PEPs*, *NEPs* and *NECGs*, was significantly suppressed in the *csl1* mutant. Considering that the *csl1* mutant exhibits a seedling lethal phenotype (Fig. 5), and that the chlorophyll content of mutant seedlings is lower than that in the WT plants (Fig. 1), suggesting that observed defects in pigmentation and chloroplast development are associated with the repression of chloroplast-associated genes [39].

## Conclusions

In summary, the findings of this study provide evidence to indicate that *OsCSL1* encodes a MAPK3 protein necessary for chloroplast development. Furthermore, *OsCSL1* may demonstrate function by regulating the expression of multiple chloroplast synthesis-related genes, thereby affecting their functions. Thus, mutation of the *OsCSL1* gene will result in wide-ranging defects, including severely disrupted chloroplasts containing accumulated starch granules and chlorotic seedlings observed in this study (Fig. 6D). Considering that most previous studies reported on MAPK cascades in plants have focused on their role in the development of immunity and elicitation of stress responses, characterization of *OsCSL1* will contribute to enhancing currently limited understanding of the essential roles of MAPKs during chloroplast development.

## Materials and methods

### Plant materials and growth conditions

The rice (*Oryza sativa* L.) *chlorosis seedling lethality 1 (csl1)* mutant, initially identified in a T-DNA insertion population with a Zhonghua11 *(japonica)* background, was kindly provided by Dr. Jingliu Zhang (Shanghai Institute of Plant Physiology and Ecology, Chinese Academy of Sciences). Over 3000 rice plants harboring T-DNA possessing a Ds element were generated via *Agrobacterium tumefaciens*-mediated transformation by Zhang and colleagues [49]. Wild-type (Zhonghua11) and *csl1* mutant plants were grown in the paddy field of South China Agricultural University, Guangzhou, China (23.16° N, 113.23 °E; subtropical climate) during the early (from late February to early July) and late (from middle July to late October).

### Insertion site analysis

Inverse polymerase chain reaction (IPCR) was performed to isolate the sequences flanking the aforementioned T-DNA region. The nested primers used for the right and left sites of the T-DNA were C1 and C2, and H1 and H2, respectively (Fig. S2). Genomic DNA digestion was initially performed using *Hin*dIII. The primers used for screening the T-DNA insertion locus were 48800+ and 5TF1 for the left site, and 49651 and 5TR2 for the right site, the sequences of which have been listed in Supplementary Table 1.

### Yeast two-hybrid (Y2H) assays

To obtain yeast competent cells, single colonies of Y2HGold (φ2–3 mm) were dispersed in 1 mL of YPDA and then transferred to a 50-mL tube containing 10 mL YPDA. Cell suspensions were incubated at 30°C for 16-18 h under shaking conditions at 250 rpm, until an OD_600_ value >1.5 was obtained. Overnight cultures were subsequently centrifuged at 3,000 rpm for 5 min at room temperature, and the resulting supernatant was discarded. Following the addition of 10 mL of fresh YPDA, the tube containing the cell pellet was vortexed vigorously for 5 min to disperse cell clumps; subsequently, incubation was performed at 30°C for 3 to 4 h under shaking conditions at 250 rpm until an OD_600_ value ranging from 0.4–0.6 was achieved. After further centrifugation at 3,000 rpm for 5 min at room temperature, the supernatant was discarded and the cell pellet was resuspended with 1.5 mL of 1× TE. To this cell suspension, 0.1 μg of each plasmid DNA and 100 μg carrier DNA were in a fresh 1.5-mL tube, followed by addition of 0.1 mL of yeast competent cells and 0.6 mL of PEG/LiAc solution (PEG3350 40%, 0.01 M Tris-HCL, 1 mM EDTA, and 0.1 M LiAc, pH 7.5). This mixture was then incubated at 30°C for 30 min under shaking conditions at 200 rpm, followed by the addition of 70 μL of DMSO and thorough mixing. The cells were then subjected to heat shock by incubating in a 42°C water bath for 15 min, followed by incubation on ice for 2 min. Following centrifugation for 30 s at 13000 rpm and room temperature, the supernatant was discarded and the pelleted cells were resuspended in 200 μL of 1× TE. Aliquots (100 μL) of this cell suspension were spread on SD/-Leu/-Trp and SD/-Leu/-Trp/-His/-Ade agar plates, respectively, followed by incubation at 30°C until the development of colonies was achieved.

### Determination of chlorophyll contents

Chlorophyll content determination experiments were conducted in accordance with the methods described by Zhang et al. (2016) [10], with slight modifications. Fresh leaf samples were chopped using scissors, and approximately 0.1 g of the cut fresh leaf material was incubated with 2 mL of extraction buffer (ethanol: propanol: H_2_O, 4.5:4.5:1 v/v/v) in a 10-mL Eppendorf tube for 12 h in the dark at 4°C, until all leaf samples presented with white coloration. Using the extraction buffer as a blank control, the maximum absorption of extracts was determined spectrophotometrically at 645 nm and 663 nm. Chlorophyll contents were calculated using the following equations: Chlorophyll *a* = (12.72 A_663_ - 2.59 A_645_) × V/W × 1000

$${\displaystyle \begin{array}{c}\mathrm{Chlorophyll}b=\left(22.88\ {\mathrm{A}}_{645}-4.67\ {\mathrm{A}}_{663}\right)\times \mathrm{V}/\mathrm{W}\times 1000\\ {}\mathrm{Total}\ \mathrm{chlorophyll}=\left(20.29\ {\mathrm{A}}_{645}+8.05\ {\mathrm{A}}_{663}\right)\times \mathrm{V}/\mathrm{W}\times 1000\end{array}}$$

### Electron microscopy

The second leaves of the wild-type and *csl1* the mutant seedlings were fixed using 2.5% glutaraldehyde for 24 h at 4°C. Thereafter, following three washes with 0.1 M PBS, the leaves were subjected to gradient dehydration using 30, 50, 70, 80, 90, and 100% acetone solution at room temperature, followed by the incubation of leaves in each concentration for 15 min. Resin penetration was then performed using an acetone: Epon812 mixture gradient (5:1, 3:1, 1:1, 1:3, and 1:5 v/v), after which the leaves were incubated in pure Epon812 for 12 h. The samples were subsequently embedded in paraffin and 60–80-nm thin sections were obtained using a microtome. The sections were subjected to staining with 2% uranyl acetate and were observed using a transmission electron microscope system (Leica EM UC6).

### RNA extraction and reversion-transcription quantitative PCR(RT-qPCR)

Total RNA extraction was conducted using the TRIzol® Reagent (Invitrogen, USA) according to the manufacturer’s instructions. First-strand cDNA synthesis from 2 μg of the extracted RNA (pre-treated with DNase I) was performed using ReverTra Ace® (TOYOBO, Japan) in 20-μL reaction systems. The reverse transcription products thus obtained were used as templates for quantitative real-time PCR (RT-qPCR) performed using the CFX ConnectTM real-time PCR system (Bio-Rad) with TB Green™ Premix Ex Taq™ II (Tli RNaseH Plus) (Takara, Japan) according to the manufacturer’s instructions. Amplification conditions were 95 °C for 2 min, followed by 40 cycles at 95 °C for 10 s and 60 °C for 30 s. Standard curves were obtained from Ct values and log concentration, and amplification efficiencies (E) were calculated according to the equation: E = 10(− 1/slope) -1 [50]. The PCR efficiency of all primers was ≥90% (Table S2), and PCR products were sequenced to evaluate primer specificity. The reference gene was selected from rice *ACTIN*, *β-TUB*, *GAPDH*, *18S-rRNA*, *UBC*, *UBQ5* and *eIF-4α*. *UBC* gene was used as a reference after being identified using geNorm software [50]. The 2^-ΔΔCt^ analysis method was used to measure the relative expression levels. Each target gene was calibrated according to the WT sample. The primer sequences used in RT-qPCR, in accordance with the MIQE guidelines [51], are listed in Supplemental Table 2.

### Plasmid construction and transformation

To generate transgenic *OsCSL1* complementation lines, a ~9-kb DNA fragment (extending from ~2.5 kb upstream of ATG to ~1 kb downstream of TGA) was amplified from ZH11 and was then cloned into a binary vector comprising a pCAMBIA2300 backbone to yield a complementary vector. To produce *OsCSL1* knockout transgenic lines, CRISPR/Cas9-induced genome editing technique was utilized. sgRNA expression cassette driven by the *Zea mays* U6a promoter was constructed, which was then assembled into a pYLCRISPR/Cas9Pubi-H vector as per methods previously described [52]. Upon verification via sequencing, the resultant vector was transformed into the *Agrobacterium tumefaciens* strain EHA105 for subsequent rice transformation. To examine the subcellular localization of the OsCSL1 protein, the pCAMBIA1300-Ubi-OsCSL1-GFP construct was generated, whereas for yeast two-hybrid (Y2H) analyses, different length segments of CSL1 and OsMKK4 were amplified using the corresponding primers. Segments of CSL1 were introduced into pGBKT7 at *Eco*RI and *Bam*HI sites, and OsMKK4 was inserted into pGADT7 subjected to digestion with *Nde*I and *Bam*HI. The corresponding primers used for amplification are listed in Supplementary Table 2.

### Subcellular localization

Rice protoplasts were isolated from 1–2-week-old rice seedlings grown on 1/2 MS medium, as per methods previously described by Zhang et al. [53] with minor modifications. The stems of seedlings were cut into 0.5-mm strips using a scalpel and were submerged in 0.6 M mannitol in the dark for 20 min. Subsequently, mannitol used herein was replaced with an enzyme solution (1.5% Cellulase RS, 0.75% Macerozyme R-10, 0.6 M mannitol, 10 mM MES at pH 5.7, 10 mM CaCl_2_, and 0.1% BSA), prepared immediately prior to use, and the stem trips were subject to digestion in the dark under shaking conditions (60 rpm) for 6 h. The protoplasts thus obtained were filtered through 40-μm nylon meshes into a 50-mL centrifuge tube and were centrifuged at 1,500 rpm to collect the protoplast pellet. After discarding the supernatant, the pellet was resuspended in 2 mL W5 solution (154 mM NaCl, 125 mM CaCl_2_, 5 mM KCl, and 2 mM MES, pH 5.7). The protoplast preparations were then transferred to several 2-mL centrifugal tubes, following which they were subjected to washing steps three times with W5 and were centrifuged at 1,500 rpm. The resultant pellet was resuspended in MMG solution (0.4 M mannitol, 15 mM MgCl_2_, and 4 mM MES, pH 5.7). For PEG-mediated transfection, 10 μg of total plasmid DNA was mixed with 100 μL of the protoplast preparation, and an equal volume of PEG solution [40% w/v PEG 4000 (Fluka), 0.2 M mannitol and 0.1 M CaCl_2_]. The mixture was then incubated in the dark for 15 min at 25°C, after which, the protoplasts were collected via centrifugation at 1,500 rpm for 3 min and were then resuspended in 1 mL of the WI solution (0.5 M mannitol, 20 mM KCl, and 4 mM MES, pH 5.7). After incubation at 25°C for 16 h, the protoplasts were examined using a confocal laser scanning microscope (Leica TCS SP8).

## Supplementary Information

**Additional file 1: Figure S1.** Chlorophyll contents and phenotypes of the wild-type and heterozygous *csl1* lines. **Figure S2.** Inverse polymerase chain reaction (IPCR) was performed to isolate sequences flanking the *OsCSL1* T-DNA*.* **Figure S3.** Comparison of the predicted (upper) and amplified (lower) OsCSL1 protein products.

**Additional file 2: Table S1.** Segregation ratio of *csl1* heterozygotes. Table S2. The primers used in this study

## Data Availability

The datasets used and/or analyzed during the current study will be available from the corresponding authors upon reasonable request.

## Abbreviations

MAPK: Mitogen-activated protein kinase
*csl1*: *Chlorosis seedling lethality 1*
ZH11: Zhonghua11
MAPK: Mitogen-activated protein kinase
MKKK22: MAPK kinase kinase22
ER: Endoplasmic reticulum
CRISPR: Clustered regularly interspaced short palindromic repeats
CBGs: Chlorophyll biosynthetic genes
PEPs: Plastid-encoded RNA polymerases
NEPs: Nuclear-encoded RNA polymerase
NECGs: Nuclear-encoded chloroplast genes
PPR: Pentatricopeptide repeat protein
TEM: Transmission electron microscopy
IPCR: Inverse polymerase chain reaction
GFP: Green fluorescent protein
Y2H: Yeast two-hybrid
