# Supplementary material for: Chlorosis seedling lethality 1 encoding a MAP3K protein is essential for chloroplast development in rice
Source: BMC Plant Biol. 2022 Jan 6;22:20. doi: 10.1186/s12870-021-03404-9 (PMC8734211; doi:10.1186/s12870-021-03404-9)
Supplement: Supplementary file 1 — Additional file 1: Figure S1. Chlorophyll contents and phenotypes of the wild-type and heterozygous csl1 lines. Figure S2. Inverse polymerase chain reaction (IPCR) was performed to isolate sequences flanking the OsCSL1 T-DNA. Figure S3. Comparison of the predicted (upper) and amplified (lower) OsCSL1 protein products. [file 12870_2021_3404_MOESM1_ESM.docx]

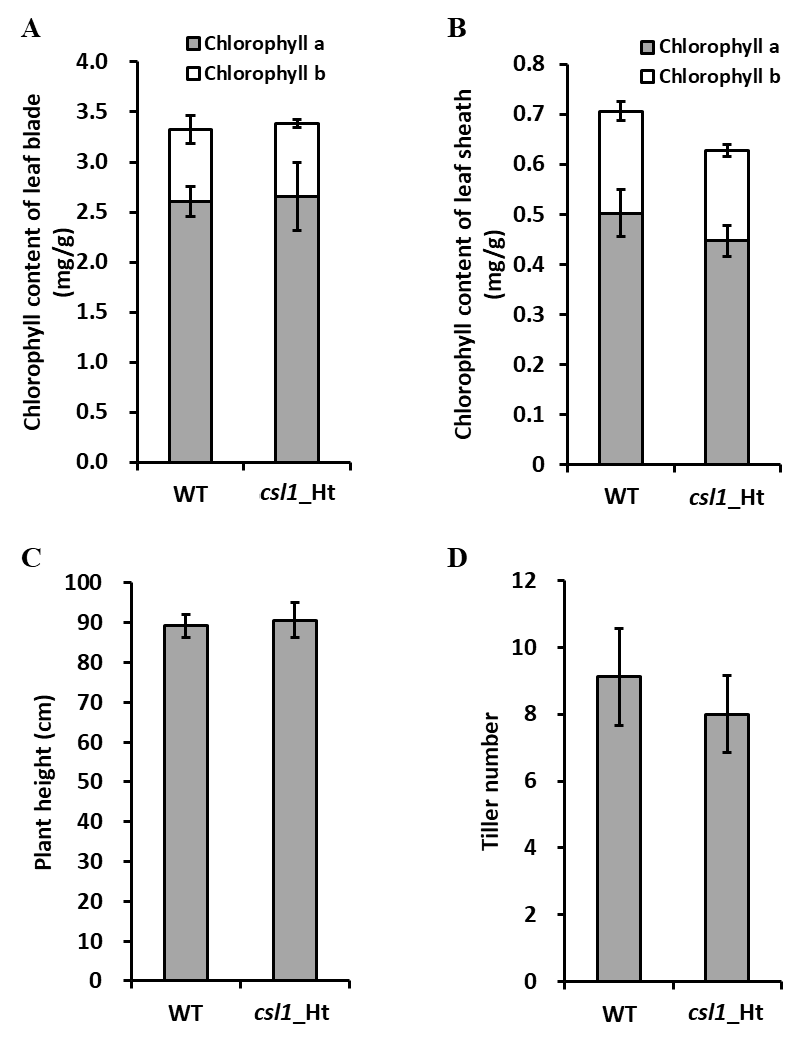


Figure S1 Chlorophyll content and phenotype between WT and *csl1* heterozygous lines. (A, B) chlorophyll content in leaf blade(A) and leaf sheath(B) in WT and *csl1* heterozygous lines; (C, D) Plant height(C) and tiller number(D) between in WT and *csl1* heterozygous lines.


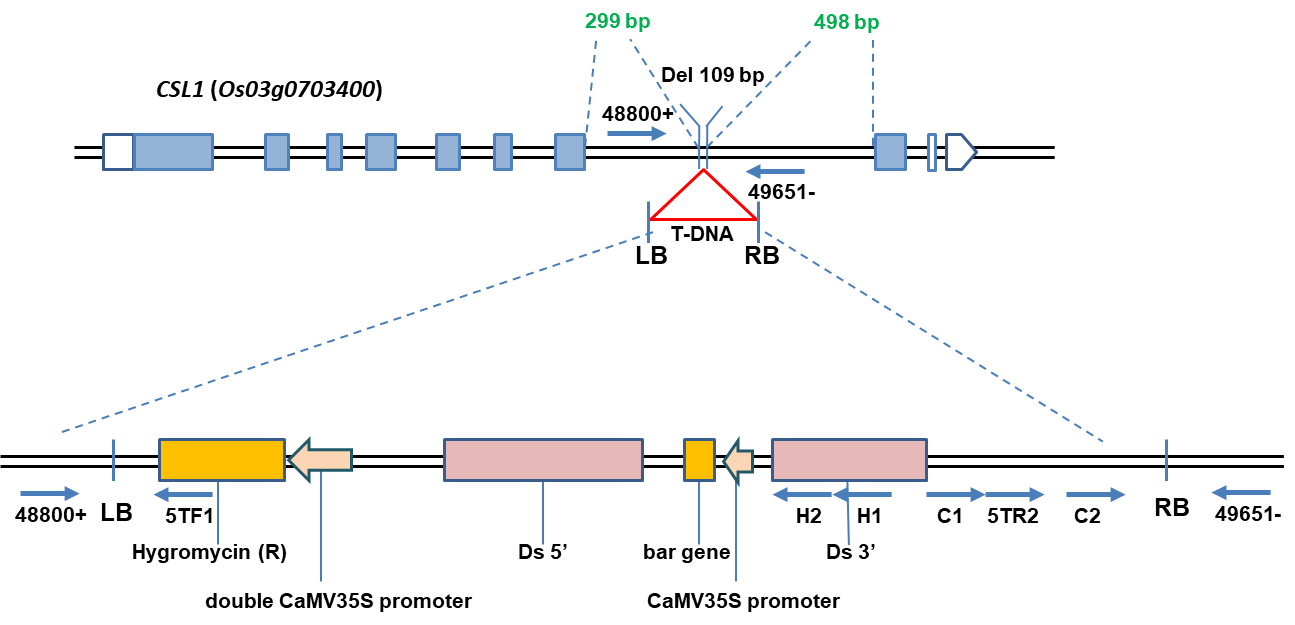


Figure S2 Inverse polymerase chain reaction (IPCR) was used to isolate the flanking sequence of T-DNA of *OsCSL1.* The nested primers of the T-DNA right border primers were C1 and C2, left border primers were H1 and H2. LB: left border; RB: right border.


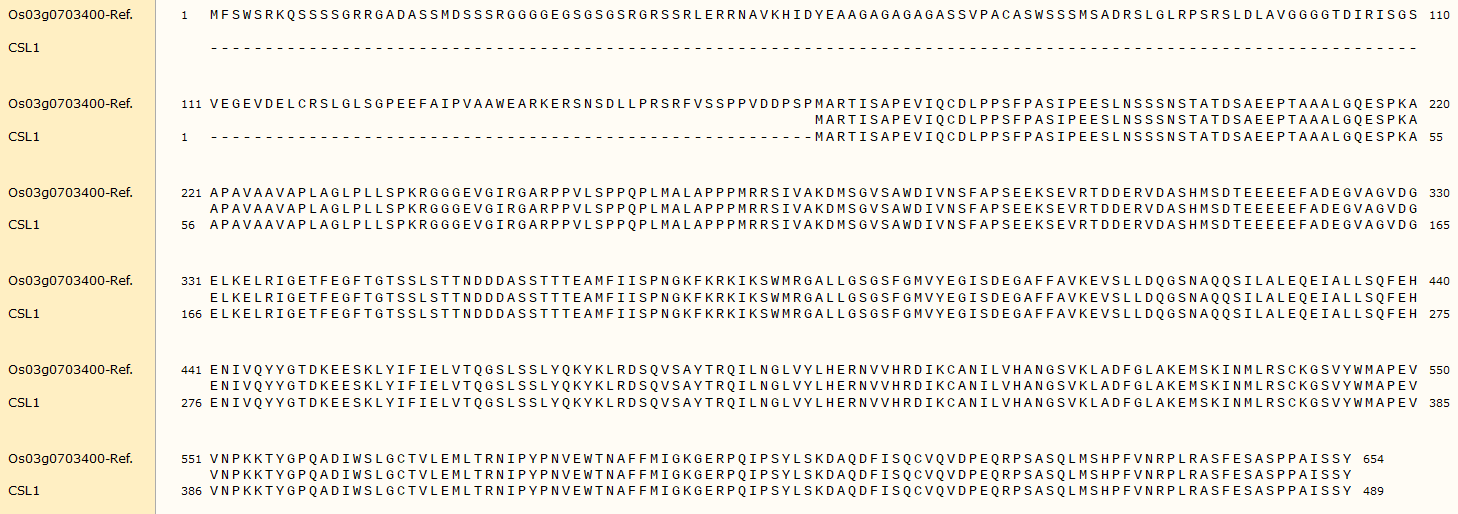


Figure S3 Comparison of OsCSL1 predicted protein product (upper) and amplified product (lower). The full-length OsCSL1 protein consists of 654aa as predicted in various databases; 165aa was missed at the beginning of first exon in the OsCSL1 amplified product.
